# Ability and Performance of Daily Activity for Older Adults With Multiple Chronic Conditions

**DOI:** 10.1093/geroni/igaa057.708

**Published:** 2020-12-16

**Authors:** Tara Klinedinst, Juleen Rodakowski

**Affiliations:** University of Pittsburgh, Pittsburgh, Pennsylvania, United States

## Abstract

Older adults with multiple chronic conditions (MCC), and especially with added depression, have lower levels of daily activity than those without MCC. Engagement in daily activities can be measured by ability (what individuals can do) and performance (what individuals actually do). Understanding this difference is critical to developing interventions that align with the daily activity needs of older adults with MCC. The aim of this study was to understand the relationships between ability and performance of instrumental activities of daily living (IADL) among older adults with varied MCC and depression status. We conducted a cross-sectional study using data from the National Health and Aging Trends Study. We used MANOVA to detect differences in ability and performance of IADL by MCC status and t-tests to test the difference between ability and performance for our sample. There was an effect of MCC status on ability and performance for older adults in this sample, F (10,13386) = 67.12, p < .001, ηp2 = .05; performance and ability were lowest for MM-D, and highest for no MCC. Post-hoc t-tests revealed a difference between the mean scores for ability (M = .79, SD = .29) and performance (M = .92, SD = .23) across all groups of MCC status, t (6693) = -50.174, p < .001. Older adults with MCC, particularly with depression, displayed diminished performance of IADL, although they have the ability. Our results suggest interventions should not only address what individuals can do, but also what they actually do in their context.

